# Parental Reminder Strategies and the Cost Implication for Improved Immunisation Outcomes: A Systematic Review and Meta-Analysis

**DOI:** 10.3390/healthcare10101996

**Published:** 2022-10-11

**Authors:** Hamina Dathini, Siti Khuzaimah Ahmad Sharoni, Kever Teriyla Robert

**Affiliations:** 1 Department of Nursing Science, Faculty of Allied Health, University of Maiduguri, Maiduguri 600104, Nigeria; 2 Centre for Nursing Studies, Faculty of Health Sciences, Universiti Teknologi MARA, Shah Alam 40450, Malaysia

**Keywords:** immunisation, parental reminder, coverage, timeliness, cost

## Abstract

Getting children vaccinated amidst prevailing barriers to immunisation has been challenging in both developed and developing countries. To address these problems, studies on parental reminder strategies were conducted to improve immunisation outcomes in children. These led to the development of different parental reminder interventions. This review systematically reviews different parental interventions and their cost implication for improved immunisations. Five online databases; Medline Complete, the Cumulative Index for Nursing and Allied Health Literature [CINAHL], Academic search premier, SPORTDiscus, and Health Source Nursing/Academic were searched using search terms. A total of 24 articles that met the inclusion criteria were included in this review. Studies that provided sufficient information were included for meta-analysis using Comprehensive Meta-Analysis version three, while narrative synthesis was used for the other studies. Results indicate that a heterogeneous and low-quality certainty of evidence on parental voice calls (OR 4.752, 95% CI 1.846–12.231, *p* = 0.001) exists in improving immunisation coverage. Regarding immunisation timeliness, a high-quality certainty of evidence on Short Message Services (SMS)-delivered health education messages (OR 2.711 95% CI 1.387–5.299, *p* = 0.004) had more effect on timely immunisation uptake. The average cost of SMS-delivered parental reminder interventions for improved immunisation outcomes was USD 0.50. The study concludes that mobile technology is a promising, cost-effective strategy for improved immunisation outcomes.

## 1. Introduction

Vaccines are either DNA recombinant, inactivated or live attenuated antigens administered to an individual to elicit an endogenous response against subsequent invasion by the pathogen; recently, blueprints of the antigen are also used [1,2]. Vaccines are effective interventions for reducing infant and child mortality [3,4]. Hence, Lwembe et al. [5] reveal that vaccination is a cost-effective global public health intervention for reducing the prevalence of infectious diseases, especially in children. In the United States, for example, children are expected to be vaccinated against 16 childhood diseases to reduce morbidity, disability, and death from infectious diseases [6].

It is estimated that over 23 million children worldwide are inadequately immunized, particularly in the first year of life when most vaccines are administered; as a result, approximately 1.5 million vaccine-preventable deaths occur [5,7]. Measles, for example, accounted for over 140,000 childhood deaths in 2018 alone, and within three years, immunisation against measles prevented over 23 million childhood deaths [8]. Accordingly, childhood vaccinations effectively prevent about 22% of death from all causes of infectious diseases in children [9]. 

In addition to preventing under-five deaths, vaccinations are a cost-effective strategy compared to the treatment cost of vaccine-preventable diseases [10]. For instance, evidence has shown that for every USD 1 spent on measles immunisation in low and middle-income countries, an estimated USD 76 is saved [10]. In this regard, Orenstein and Ahmed [11] reported a return on investment of USD 586 billion for every USD 34 billion of direct costs of vaccination in developing countries, with a net direct and indirect cost of USD 1.53 trillion. This is huge. Similarly, in a high-income country, the United States, an estimate shows that the immunisation program saved about USD 69 billion in direct and indirect costs [6].

Active parental involvement in immunisation services is one of the most significant challenges to adequate immunisation coverage [11]. Several factors impede the active involvement of parents in immunisation services, they are negligence, acceptance, attitudes and forgetfulness [9,11]. Of these factors, evidence suggests that forgetfulness of scheduled vaccination appointments is the most prevalent of the impeding factors [12,13]. To address these problems, studies on parental reminder strategies were conducted to find an effective strategy to motivate parents to vaccinate their children. These led to the development of different parental reminder interventions. It is therefore paramount to systematically review these studies to determine the most effective strategy for improved childhood immunisation outcomes.

There are a few systematic reviews conducted in this regard; some were based on the effect of parental reminders in developing countries [14,15], others were conducted a long time ago, and new evidence has emerged [16] or narrowed to a specific intervention [15,17,18]. Others targeted adolescent or adult populations, not children under five [3,7]. Therefore, studies conducted in high- and low-income countries need to be evaluated as there is new evidence and to systematically review the cost implications of delivering the parental reminder strategies. Hence, this systematic review could provide evidence of different parental reminder strategies and cost implications for enhanced coverage and timeliness of immunisation.

### Objectives

- To determine the effective parental strategy for improving coverage of childhood immunisation;
- To determine the effective parental strategy for enhancing the timeliness of childhood immunisation;
- To determine the cost associated with delivering parental strategies for improved immunisation uptake.

## 2. Materials and Methods

The Preferred Reporting Items for Systematic reviews and Meta-Analysis [PRISMA] was developed and introduced by Cochrane in 2009 to ensure consistency in the reporting of systematic reviews [19]. However, the PRISMA was updated and expanded in 2020 to overcome the limitations of the 2009 PRISMA [20]. Therefore, the 2020 version of PRISMA was adopted for this review. The PRISMA 2020 checklist can be accessed at https://prisma-statement.org/PRISMAStatement/Checklist.aspx (accessed on 29 August 2022).

### 2.1. Eligibility Criteria

The inclusion and exclusion criteria used for this review is as shown on Table 1 below.

### 2.2. Information Sources

The systematic search for articles was conducted from Wednesday 29 June to Thursday 7 July 2022. Articles searches were conducted using five online databases: Medline Complete, the Cumulative Index for Nursing and Allied Health Literature [CINAHL], Academic search premier, SPORTDiscus and Health Source Nursing/Academic.

### 2.3. Search Strategy and Selection Process

The search for articles was performed using appropriate MeSH and keywords in Medline Complete, CINAHL, SPORTDiscus, Health Source Nursing/Academic and Academic search premier. Boolean operators such as “AND” and “OR” and wild cards “*” were used in addition to search terms for search in the databases. The search terms used are: (‘‘parental reminder’’ OR ‘‘reminder system*’’ OR ‘‘retrieval strateg*’’ OR ‘‘recall initiative*’’) AND (monitor* OR improv* OR impact* OR affect* OR effect* OR determin* OR assess* OR evaluat* OR measur* OR influenc*) AND (‘‘immunization’’ OR ‘‘immunization program*’’ OR ‘‘immunization status’’ OR ‘‘immunization level’’ OR ‘‘immunization performance’’). Article search was limited to online databases of articles published between 2015 to 2022 to enable researchers to find current evidence on the strategies investigated on parental reminders for enhanced immunisation outcomes. The reference list of the systematic review was also performed to identify literature that might have been missed.

### 2.4. Study Selection

The Prisma template guided study selection. The databases were synchronously searched using Ebscohost. The system automatically removes duplicates. Subsequently, the inclusion and exclusion criteria of the study guided the screening of the title and abstract. After that, full-text screening was conducted to determine eligible studies. The study selection was independently made by two researchers (D.H. and S.K.A.S.); where there were disagreements, a consensus was used to resolve them. 

### 2.5. Data Collection Process

The data collection process of the eligible studies was performed independently by D.H. and verified by S.K.A.S. using spreadsheets.

### 2.6. Data Items

Items to be extracted from studies are critical for a successful review, and it is therefore essential for researchers to carefully determine items needed for the systematic review and conscientiously extract them [21]. The information extracted for this review includes the author, year of publication, study participants, sample size, intervention(s) for each intervention category, comparator and their sample size, research design, vaccine type and country. Others include the dose of the intervention, which includes the frequency and course of the intervention, outcomes, instrument for data collection and results. Two researchers were involved in the data extraction process to minimize errors during the data extraction. Firstly, D.H. performed the initial data extraction, and S.K.A.S. verified to ensure the correct data were extracted. The table of data extraction is presented in Table A1.

### 2.7. Risk of Bias in Individual Studies

The selected 24 articles that met the inclusion criteria were assessed for risk of bias. Two study designs were generally included in the review, randomized control trials and quasi-experimental studies. Due to the two types of studies included in the review, the Physiotherapy Evidence Database Scale (PEDro scale) and Crowe Critical Appraisal Tool (CCAT) were used for bias assessment for randomised control trials (RCT) and quasi-experimental studies, respectively. They were chosen because of their good validity, reliability and acceptability across the globe as an easy-to-use tool with reliable and meaningful practical application [22]. The PEDro scale has 11 items scored as either Yes = 1 point or No/not sure = 0 [23]. Item one denotes the study’s eligibility [24]. The score is obtained by summing items 2 to 11 and assessed out of 10, while the internal validity is obtained by summing items 2 to 9 marked out of 8. Lastly, the reporting subscale is obtained by adding items 10 and 11, rated out of 2 [24]. Interpretation of the PEDro scale is 0–3 = poor, 4–5 fair, 6–8 good and 9–10 = excellent. The higher the score, the better the article. For this study, any article that ranges from a score of 4 (fair) to 10 (excellent) was included [23]. Table 2 shows the summary findings of the risk of bias of all RCTs included in this review. On the other hand, quasi-experimental studies included in the review were assessed using CCAT. The CCAT assesses eight components of a research article and is scored from zero to five. The total score is obtained by summing items one to eight and is scored out of 40. The final score is then converted and presented as a percentage by dividing the total score by 40 and multiplying by 100 [25]. Although Crowe left open the interpretation of the score to the assessor to make based on individual study requirements, Salihu et al. [24], in their review, revealed a score of <50 as poor and >50 as good for the study to be rejected or accepted accordingly. This review hence adopted it. Table 3 shows the risk of bias in the studies using the CCAT. Two independent reviewers performed the quality appraisal, D.H and S.K.A.S., a third independent reviewer resolved disparity.

**Table 2:** Quality appraisal of RCT articles using the PEDro scale.

Author	Year	Eligibility	Randomised Allocation	Concealed Allocation	Similarity at Baseline	Blinding of Participants	Blinding of Therapist	Blinding of Assessor	Dropout	Intention to Treat	Group Comparison	PMVD	Total Score (10)	Internal Validity (8)	SUBSCALE (2)	Interpretation	Decision
Nagar et al.	[7]	Yes	Yes	Yes	Yes	No	No	No	Yes	Yes	Yes	Yes	7	5	2	Good	Accepted
Mekonnen et al.	[9]	Yes	Yes	Yes	Yes	No	Yes	Yes	Yes	Yes	Yes	Yes	9	7	2	Excellent	Accepted
Wallace et al.	[12]	Yes	Yes	No	Yes	No	No	Yes	No	Yes	Ye s	Yes	6	4	2	Good	Accepted
Niederhauser et al.	[26]	Yes	Yes	No	Yes	No	No	No	Yes	Yes	Yes	No	6	4	2	Good	Accepted
Busso et al.	[27]	Yes	Yes	No	Yes	No	No	No	Yes	Yes	Yes	Yes	6	4	2	Good	Accepted
Kempe et al.	[28]	Yes	No	No	Yes	No	No	No	Yes	Yes	Yes	Yes	5	3	2	Fair	Accepted
Bangure et al.	[29]	Yes	Yes	Yes	Yes	No	No	No	Yes	Yes	Yes	No	6	5	1	Good	Accepted
Brown et al.	[30]	Yes	Yes	No	Yes	No	No	No	Yes	Yes	Yes	Yes	6	4	2	Good	Accepted
Gibson et al.	[31]	Yes	Yes	Yes	Yes	No	No	Yes	Yes	Yes	Yes	Yes	8	7	1	Good	Accepted
Seth et al.	[32]	Yes	Yes	Yes	Yes	No	No	No	Yes	Yes	Yes	Yes	7	5	2	Good	Accepted
O’Grady et al.	[33]	Yes	Yes	Yes	Yes	No	No	Yes	Yes	Yes	Yes	Yes	8	6	2	Good	Accepted
Domek et al.	[34]	Yes	Yes	Yes	Yes	No	Yes	Yes	Yes	Yes	Yes	No	8	7	1	Good	Accepted
Menzies et al.	[35]	Yes	Yes	Yes	Yes	No	No	No	Yes	Yes	Yes	Yes	7	5	2	Good	Accepted
Brownstone	[36]	Yes	Yes	No	Yes	No	No	No	Yes	No	Yes	Yes	5	3	2	Fair	Accepted
Siddiqi et al.	[37]	Yes	Yes	Yes	Yes	No	No	No	Yes	Yes	Yes	Yes	7	5	2	Good	Accepted
Kagucia	[38]	Yes	Yes	Yes	Yes	No	No	No	Yes	Yes	Yes	Yes	7	5	2	Good	Accepted
Kazi et al.	[39]	Yes	Yes	Yes	Yes	No	No	No	Yes	Yes	Yes	Yes	7	6	2	Good	Accepted
Ekhaguere et al.	[40]	Yes	Yes	Yes	Yes	No	Yes	No	No	Yes	Yes	Yes	7	5	2	Good	Accepted
Dissieka et al.	[41]	Yes	Yes	Yes	Yes	No	No	No	No	No	Yes	Yes	5	3	2	Fair	Accepted
Hofstetter et al.	[42]	Yes	Yes	Yes	Yes	No	No	No	Yes	Yes	Yes	Yes	7	5	2	Good	Accepted

Note: PMVD = Point measures and variability data.

**Table 3:** Crowe critical appraisal for quasi-experimental studies.

No	Author	Year	Preliminary	Introduction	Design	Sampling	Data Collection	Ethical Matter	Results	Discussion	Total Score	% Score	Decision
1	Oladepo	[13]	4	4	4	3	3	4	4	4	30	75	Accepted
2	Yunusa	[15]	3	4	4	3	4	4	4	3	29	73	Accepted
3	Uddin et al.	[43]	4	3	4	4	4	4	3	4	31	78	Accepted
4	Ibraheem et al.	[44]	4	3	4	3	4	3	4	4	29	73	Accepted

Salihu et al. [24] reveal a score of <50 as poor and >50 as good for the study to be rejected or accepted accordingly.

### 2.8. Certainty of Evidence

Certainty of evidence was assessed using the GRADEpro (Grading of Recommendations Assessment, Development and Evaluation) Tool for all the included studies. The funnel plots were used to help in assessing reporting bias.

### 2.9. Data Analysis

Meta-analysis was conducted for studies that provide sufficient information for inclusion. The original data used for the meta-analysis were a proportion of their respective outcomes. The studies were analyzed using Comprehensive Meta-Analysis software (CMA) version three. The random effect model with odds ratios was used as the pool size measurement. A *p*-value < 0.05 shows a result as statistically significant. The random effect model was used because of the expected level of heterogeneity of some of the included studies. Additionally, the heterogeneous spread of the odds ratio between research studies was determined by calculating the Q-statistic and I^2^ to represent the heterogeneous variations between studies expressed as a percentage. In order to interpret the level of significance for clinical decision-making, Hopkin’s scale, a table of effect size and their levels of significance was used [45,46]. See Table A2 for the complete table of effect sizes and their interpretation. In studies that were not analyzed using meta-analysis, a narrative analysis was performed using themes and sub-themes.

## 3. Results

### 3.1. Study Selection

**Figure 1:**
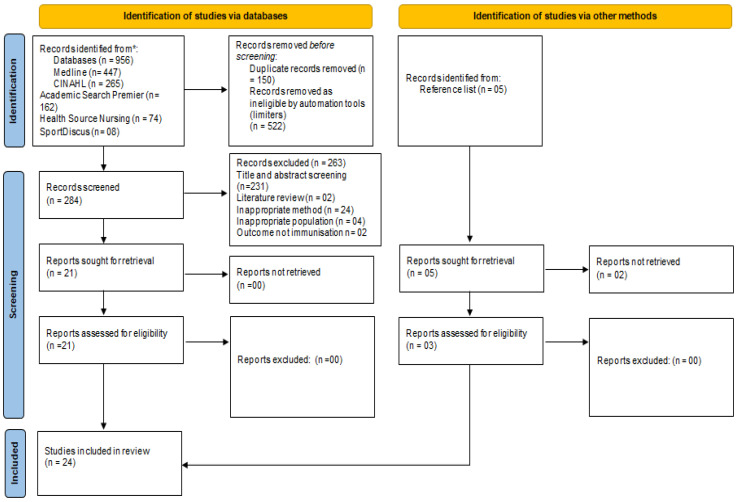
PRISMA flow chart.

The search was conducted in five online databases and turned out the following articles: Medline complete (447), CINAHL (265), SPORTDiscus (08), Health Source Nursing/Academic (74) and Academic search premier (162). A total of 956 research articles were retrieved through online databases. Subsequently, limiters were applied to narrow the search to the desired requirements. Limiters applied were: only studies conducted in English due to the inability of the researchers to interpret and time constraints to get an interpreter, only studies conducted in the past seven years, i.e., 2015 to 2022, to allow for only current pieces of evidence to be included in the review. Another limiter used was only research articles published in academic journals. After applying the limiters, a total of 522 articles were removed. Research articles were further narrowed by 150 after duplicated studies were removed. Hence, 284 articles were left for screening. The screening was conducted based on title, abstract and subsequently, full-length reading. Two hundred thirty-one articles were removed after the title and abstract reading and 32 after full-length, leaving 21. To search for grey literature, reference lists were searched and a total of five new articles were found, however, only three were able to be retrieved, hence, a total of 24 articles were available for quality appraisal. The screening for articles was performed by D.H. and independently verified by S.K.A.S. The 24 articles selected after full-length reading were subjected to quality appraisal to determine their validity. All 24 articles were accepted for inclusion in the review. Figure 1 shows the PRISMA flow chart of the screening and inclusion processes of the articles.

### 3.2. Characteristics of Included Studies

Overall, 24 studies that met the inclusion criteria were used for this review. A total of 54,224 parents were recruited as participants in all the studies and numbers ranged from 42 in the study of Niederhauser et al. [26] to 13,000 in the study of Busso et al. [27]. In almost all the studies, participants were reportedly mothers or caregivers of the children [7,9,12,13,15,27,28,29,30,31,32,33,34,35,36,37,38,39,40,41,43,44]. Two studies reportedly mentioned males as participants, Hofstetter et al. [39] reported a nearly even distribution of participants, male (1051) and females (1003) and Niederhauser et al. [26] reported only one male participant in their study. 

Regarding the categorisation of the countries of the included studies, the World Bank income classification reveals that 5 studies [26,28,33,35,42] were conducted in high-income countries, while the remaining 19 were conducted in low- or middle-income countries. Additionally, based on the study’s inclusion criteria, 4 studies [13,15,43,44] were quasi-experimental, while the remaining 20 were randomized control trials. Of the 20 RCTs, 3 [30,31,41] were cluster randomized trials. The course of the intervention ranges from 3 months in the study of Kagucia et al. [38] to 16 months in the study of Hofstetter et al. [42]. Mobile phone Short Message Services are the most common intervention for all the studies. Details of the studies are presented in Table A1.

### 3.3. Risk of Bias

The risk of bias in the studies was assessed using two different methods based on their designs. PEDro and CCAT were used for RCTs and quasi-studies, respectively. A summary of the risk of bias of the included 20 RCT studies indicates that most of the studies were rated as good quality—16 (80%), 14 (70%) studies clearly described their concealment of allocation and in the majority of the studies, blinding could not be achieved. Almost all—18 (90%)—of the studies reported analyzing results using intention to treat. Quasi-experimental studies were all found to be of acceptable quality. The total score ranges from 73% [15] to 78% [43]. Refer to Table 2 and Table 3 for a detailed assessment of the risk of bias.

### 3.4. Findings from Meta-Analysis

Twenty-four studies were included in the review to evaluated parental reminder or recall strategies for improved immunisation. Out of the 24 studies, 19 [9,13,15,26,29,30,31,32,33,34,35,36,38,39,40,41,42,43,44] provided sufficient information and were therefore included for meta-analysis. The 19 studies assess five different parental reminder/recall strategies on two major immunisation outcomes: coverage and timeliness of immunisation among children.

#### 3.4.1. Coverage of Immunisation

**Figure 2:**
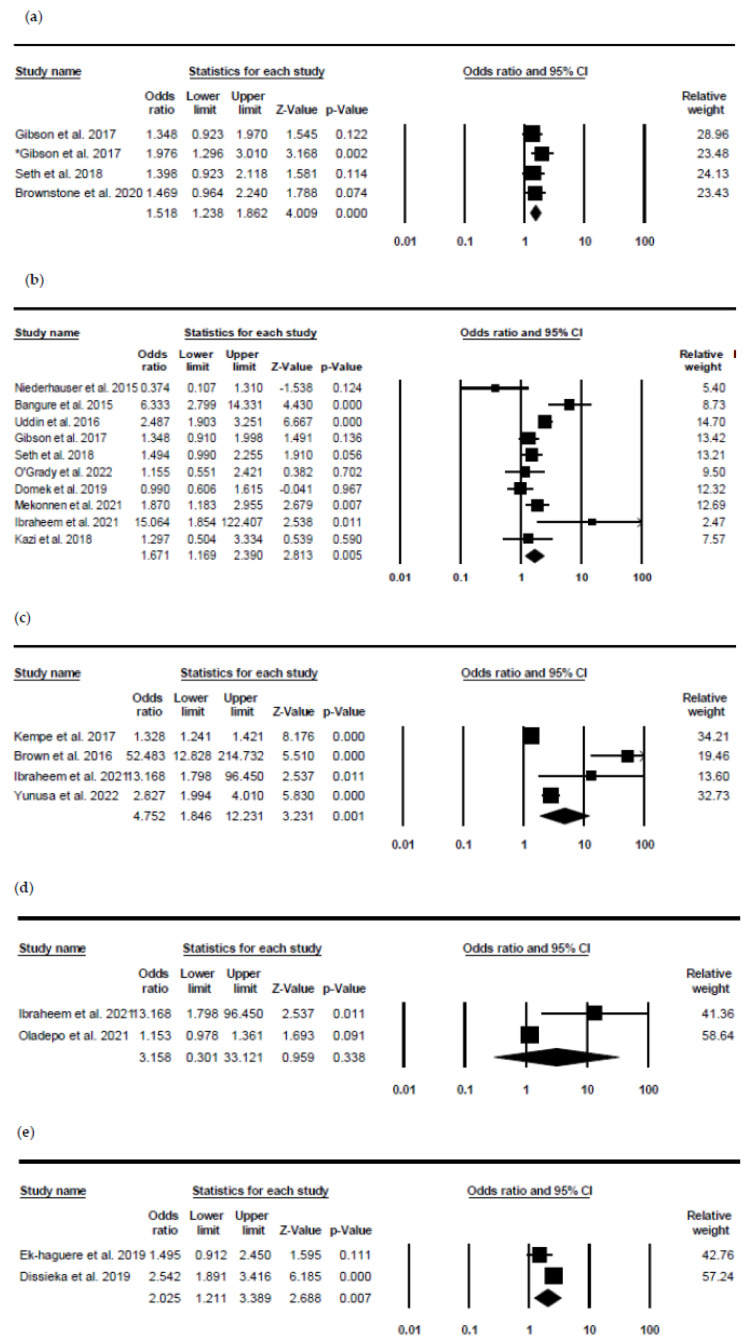
Forest plot of interventions on immunisation coverage. (**a**) SMS reminder and incentive on coverage: Heterogeneity: Q-value = 2.051, *p*-value = 0.562, I-square 0.000 [31,32,36]. (**b**) Reminder SMS on coverage. Heterogeneity: Q = 35.13, *p* = 0.001, I-square = 74.381 [9,26,29,31,32,33,34,39,43,44]. (**c**) Voice call on coverage. Heterogeneity: Q-value = 47.973, *p*-value = 0.001, I-square 93.746 [15,30,32,42]. (**d**) SMS health education. Heterogeneity: Q-value = 5.705, *p*-value = 0.017, I-square 82.472 [13,44]. (**e**) Voice call and SMS on coverage. Heterogeneity: Q = 3.265, df = 1, *p* = 0.071, I2 = 69.37 [40,41].

Figure 2 below shows the results of the meta-analysis of five different intervention on the coverage of immunisation in children. The interventions are SMS reminder and incentive, reminder SMS, Voice call, SMS delivered health education and Voice call with SMS reminder. 

#### 3.4.2. Timeliness of Immunisation

**Figure 3:**
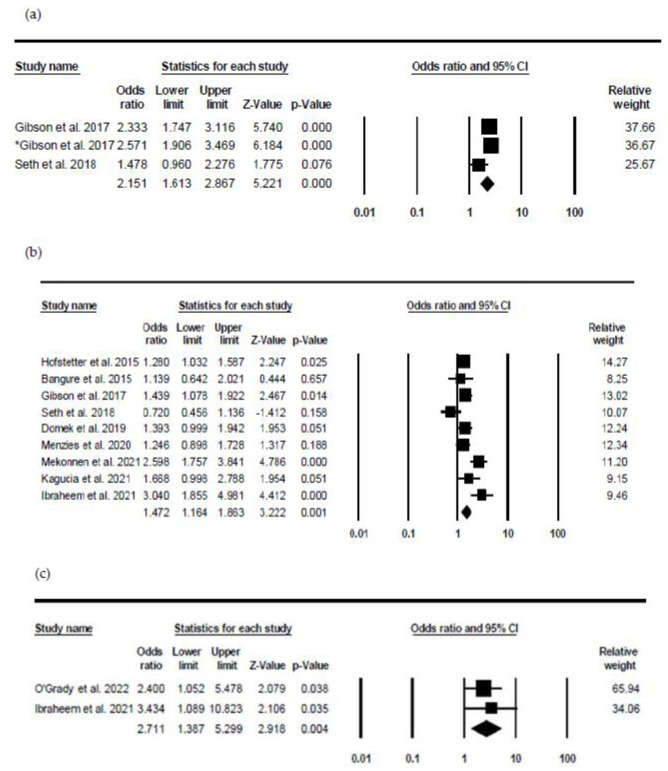
Forest plot of interventions on timeliness. (**a**) SMS reminder and incentive on timeliness. Heterogeneity: Q-value = 4.453, *p* = 0.108, I-square 55.089 [31,32]. (**b**) SMS reminder. Heterogeneity: Q-value = 29.290, *p* = 0.001, I-square = 72.687 [9,29,31,32,34,35,38,42,44] (**c**) SMS health education on timeliness. Heterogeneity: Q-value = 0.246, *p* = 0.620, I-square = 0.000 [33,44].

Figure 3 below shows the results of three different interventions on the timeliness of immunisation. The interventions are SMS reminder with incentive, SMS remiders and SMS delivered health education messages.

Three studies were found to provide sufficient information and analysed using meta-analysis regarding the influence of incentives on improving immunisation coverage. However, Gibson et al. [31] appeared twice because the study assessed the difference between two incentives for improved immunisation outcomes against the control, hence, making *n* = 4. The finding from the study indicates an odds ratio (OR) of 1.5 95% CI 1.238, 862, *p* ≥ 0.001, *n* = 4. With regards to the heterogeneity, the I^2^ was 0.000 indicating an absence of heterogeneity. It is important to note that all the studies are rated using PEDro to be of good quality except the study of Brownstone et al. [36], which was found to be of fair quality.

Regarding the influence of SMS reminders in improving immunisation coverage, ten studies were found to have sufficient information for meta-analysis. Of the ten studies, two were from high-income countries [26,33], and the remaining studies were from low- and middle-income countries. It is important to note that the studies were all rated as having “good” quality except Mekonnen et al. [9], which was found to be of excellent quality. The pooled effect of parental SMS reminder for improved immunisation coverage was (OR = 1.671, 95% CI 1.169, 2.390, *p* = 0.005, *n* = 10). This shows that SMS intervention is approximately 1.7 times more likely to improve immunisation coverage compared to the control group because the p-value is less than 0.05. The I^2^ was found to be 74.781 indicating large heterogeneity exists in the studies based on the rule of thumbs.

A significant effect was found on the effect of voice calls in improving immunisation coverage, the pooled effect of four studies analysed were (OR 4.752, 95% CI 1.846, 12.231, *p* = 0.001, *n* = 4). Voice call intervention was about five times more likely to improve immunisation coverage compared to the control group with standard practice. It is worth noting that the study of Kempe et al. [28] was of “fair quality”. Although the remaining three studies are of “good” quality, they are all conducted in the same country, Nigeria, a low- and middle-income country; hence its generalisation should be used with caution. Additionally, the I^2^ (93.746) indicates that a large level of heterogeneity exists.

With regards to the effectiveness of health education messages delivered through mobile SMS on immunisation coverage, a large level of heterogeneity was identified with the I^2^ value of 82.472. The pooled effect (OR 3.158, 95% CI 0.301, 33.121, *p* = 0.338, *n* = 2) of the meta-analysis shows that intervention improves immunisation coverage by 3.2 times compared to the control group. Although the intervention was found to have odds of about 3.2, it is not statistically significant because the *p* = value of 0.338 is greater than 0.05.

Lastly, the pooled effect of a combination of voice calls and SMS reminders shows that intervention improved coverage of immunisation with the odds of 3.025 compared to the control group. The pooled effect revealed (OR 3.025, 95% CI 1.211, 3.389, *p* = 0.007) and the I^2^ of 69.37 indicates that a moderate level of heterogeneity exists.

The effect of incentives on timely immunisation uptake was found to be statistically significant with the pooled effect of (OR 2.151, 95% CI 1.613, 2.867, *p*-value = 0.001). This means incentives can improve timely immunisation uptake by 2.1 times compared to standard routine care. The study of Gibson et al. [31] appeared twice because it assessed the effectiveness of two different (75KES and 200KES) incentives against control on the timeliness of immunisation. The heterogeneity level was found to be moderate as the I^2^ value was 55.089. 

In assessing the effectiveness of parental reminder SMS on timely immunisation uptake, nine provided sufficient information and were analysed using meta-analysis. The heterogeneity as reported by I^2^ (72.687) was interpreted to be moderate based on the rule of thumb. The pooled effect of the results indicates that intervention was statistically significant and about 1.5 times more likely to improve timely immunisation uptake than the control group (OR 1.472 95% CI 1.164, 1.883, *p* = 0.001, *n* = 9). The quality assessment of the studies indicates that one [9] was assessed to be of excellent quality based on the PEDro tool, while the remaining studies are rated as good quality.

Lastly, the effect of health education messages on timely vaccination shows the following pooled size effect (OR 2.711 95% CI 1.387, 5.299, *p* = 0.004). As the *p*-value of 0.018 is less than 0.05, health education messages delivered through SMS are statistically significant in improving the timely vaccination of children. Additionally, because of OR 2.711, participants in the intervention group are likely to be 2.7 times timely to have their infants vaccinated on time compared to those in the control group. The I^2^ of the 0.000 was also reported indicating the absence of heterogeneity in the included studies. 

### 3.5. Narrative Synthesis

Four studies [7,12,27,37] used different forms of interventions in assessing immunisation coverage and timeliness in their studies and therefore were narratively analysed. Of the four studies included in this section, all reported outcomes on immunisation coverage, while only two [12,37] reported outcomes on the timeliness of vaccination.

#### 3.5.1. Coverage of Immunisation

The study of Busso et al. [27] targets a community outreach program conducted once a month for six months and shows a positive effect on immunisation coverage by increasing coverage of immunisation among the population by up to 4.6% when compared with untargeted community outreach. Another study conducted in India by Nagar et al. [7] assessed immunisation coverage between three different interventions. The effectiveness of using a pendant plus one voice call a day to the immunisation due date, pendant alone or the NFC stickers placed on the child’s home-based record were assessed. The results were 69.4, 57.4 and 58.7% for pendant with voice call, pendant only and NFC stickers, respectively, at the endpoint. This indicates that the use of a pendant with voice call can lead to an increase in immunisation coverage compared with an NFC sticker, with a percentage difference of 10.7 per cent between the pendant plus voice call group and the NFC sticker group. Additionally, Wallace et al. [12] compared the use of home-based records alone, home-based records with stickers and standard care on immunisation coverage. Results indicate that home-based record plus sticker group for DTPcv3 vaccination completion rate was (77%) compared with the control group (RR = 0.97, 95% CI: 0.90, 1.04), and HBR-only group for DTPcv3 (74%) compared with the control group (RR = 0.94, 95% CI: 0.87, 1.02). HBR + sticker vs. control (77 vs. 78%) (RR = 0.99, 95% CI: 0.98, 1.09), HBR-only vs. control 74% (RR = 0.96, 95% CI: 0.88, 1.05). These results show that both interventions were not statistically significant in improving immunisation coverage. Lastly, Siddiqi et al. [37] compared the effectiveness of two bracelets in improving immunisation coverage in Pakistan; the Alma Sana Bracelet and the Star Bracelet. Results of the interventions reveal that interventions were both effective but not significant for improved coverage of immunisation coverage of Penta 3 at 12 months is 84.3, 85.4 and 83.0% for Alma Sana Bracelet, Star Bracelet and the control group, respectively. While measles coverage at 12 months was 72.0, 70.5 and 68.5% for Alma Sana Bracelet, Star Bracelet and the control group, respectively. Although using culturally appropriate intervention was novel, it did not bring about significant change compared to the control group.

#### 3.5.2. Timeliness of Immunisation

Regarding the timeliness of immunisation, two studies [7,12] were used to narratively analyse the impact of interventions on the timeliness of vaccination. In the study of Nagar et al. [7], the use of a pendant with voice call led to 69.4% of the study participants’ timely immunisation uptake compared to 58.7% in the NFC stickers of the control group. On the other hand, when the pendant was used alone, there was no significant difference in its use (57.4%) compared with the use of NFC stickers (58.7%). This could then be implied that the use of voice call in the pendant with voice call group brought about the impact witnessed in that study arm group. In the study of Wallace et al. [12], the timeliness of vaccination within 60 days for the home-based record plus sticker group was 32%, for the home-based record was 24%, and for the control, 23%. The percentage difference between the intervention groups compared to the control group was +8% and +9% for home-based record plus sticker and home-based record groups, respectively. 

### 3.6. Cost Implication

The cost implications for implementing interventions were also reported in four studies [27,28,29,32]. The cost per child ranges from USD 0.0075 in the study of Ek-haguere et al. [40] to USD 10 in the study of Kempe et al. [28]. Only Kempe et al. [28] reported the cost implication in the developed country, while the remaining three studies [27,29,32] were from low- and middle-income countries. From the reports, the average cost of implementing a collaborative centralised call was USD 10. In developing countries, two interventions on SMS reminder cost ranges from USD 0.0075 to USD 0.99, which is an average of USD 0.50 per child. Targeted outreach was reported to cost USD 0.11 per additional child [27] while voice call message cost USD 0.015 per child [40].

### 3.7. Certainty of Evidence

**Figure A1:**
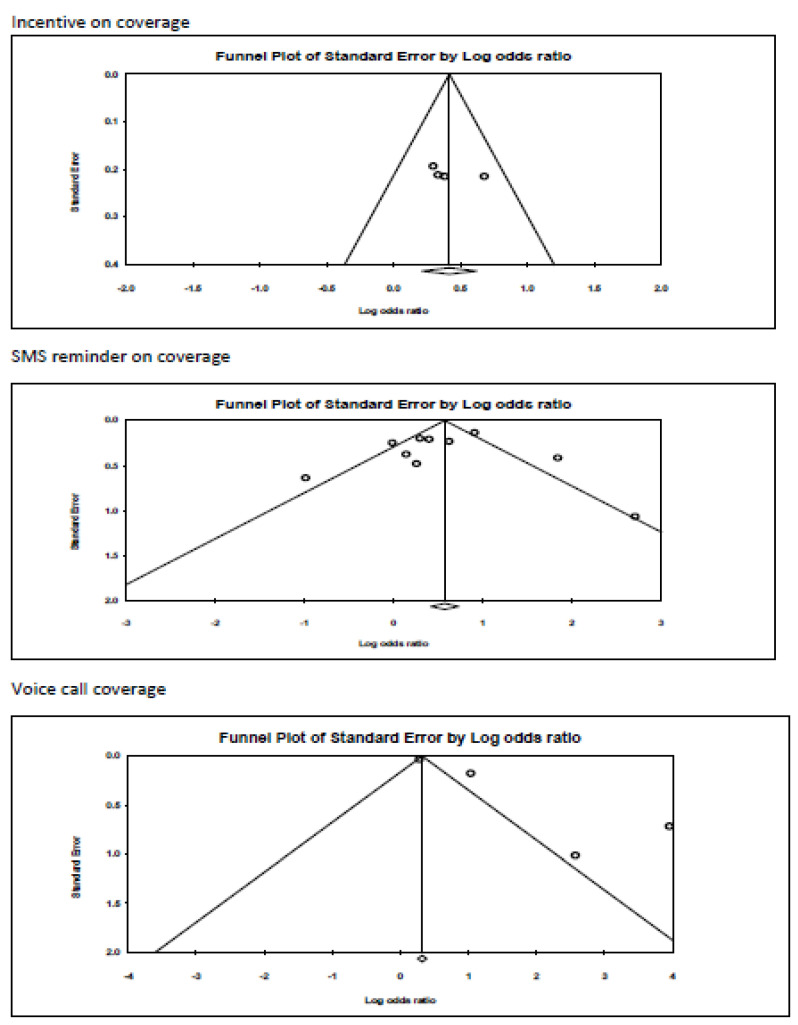
Funnel plots on coverage.

**Figure A2:**
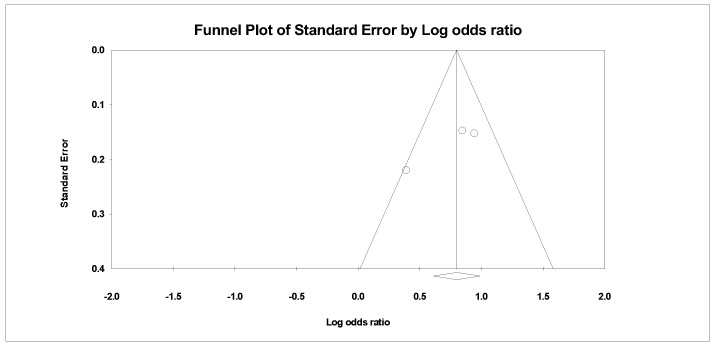
SMS reminder timeliness.

**Figure A3:**
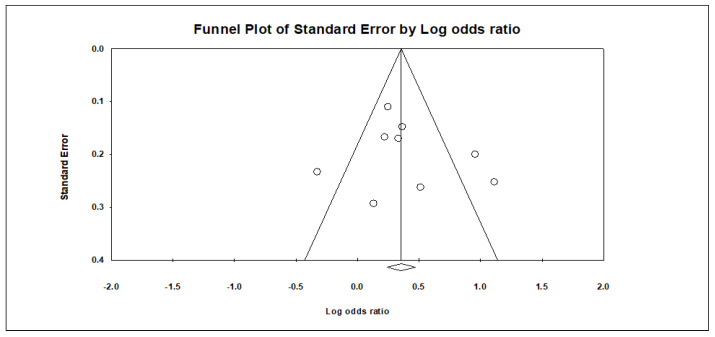
SMS reminder on timeliness.

Certainty of evidence was assessed using GRADEpro, result of the assessment indicates that certainty of the evidence for interventions on coverage of immunisation ranges from very low (SMS reminder) to moderate (incentive; voice call with SMS reminder). For timeliness of immunisation, certainty of evidence was moderate (incentive), very low (SMS reminder) and high (SMS health education). The detail of the assessment of certainty of evidence is shown in Table 4 and Table 5. For reporting bias, outcomes that could be assessed using the funnel plots were retrieved and visualised for reporting bias. The funnel plots are in Figure A1, Figure A2 and Figure A3.

## 4. Discussion

To address the persistently low coverage of immunisation, this review assessed the effect of parental strategies on improved immunisation outcomes. Twenty-four studies that met the inclusion criteria were at low risk for bias. The total number of parent–child pairs in the included studies was 54,224. Most of the studies were from low- and middle-income countries. This disparity in study location could be attributed to the fact that most hesitancy to vaccination is due to a lack of awareness of vaccine importance, mostly in low- and middle-income countries and the ceiling effect in high-income countries [47]. This might be the rationale that spurs more studies in developing compared to developed countries. Thirteen different parental strategies were assessed across the 24 included studies to determine the effect of such intervention on three key outcomes. The outcomes assessed were immunisation coverage, timeliness, and the cost implication of the interventions. The effects of these interventions are discussed under these themes.

Building a vaccination culture within the healthcare system and critical stakeholders such as parents is fundamental for improved outcomes and safe and effective vaccine delivery [48]. Over the years, different strategies have been adopted to improve immunisation coverage across the globe. Five interventions (mobile voice call, SMS reminder, use of incentives, SMS health education and a combination of voice call with SMS reminder) were evaluated using meta-analysis to determine the most effective strategy for improved immunisation coverage. Finding reveals that all interventions were statistically significant for improving immunisation coverage except for SMS health education. Although the pooled effect of SMS health education messages was found to have improved immunisation coverage among the study participants, it was not statistically significant. Therefore, to improve immunisation coverage, the meta-analysis results reveal that voice call produced the largest effect in improving immunisation coverage. The use of mobile phone voice calls was found to be approximately five times most likely to improve immunisation coverage compared to the control participants, and this effect was statistically significant. To determine how significant the intervention was, evidence has recommended using Hopkin’s scale for determining effect size in the odds ratio category, and an odds ratio of 4.752 is interpreted to have a moderate effect [45]. Hence it can be said that mobile voice calls to parents can moderately improve immunisation coverage. The certainty of evidence is rated low as such; the assessed result may be considerably different from the true effect. Additionally, large heterogeneity exists, this could be due to the differences in the included studies as seen in the wide dispersion of the confidence interval and the relative weight of the studies. Furthermore, this heterogeneity could be related to sampling error or low retention rate. Due to these reasons and the fact that three [15,30,44] of the four studies used to determine these effects were from one country (Nigeria); its interpretation and use should be performed with caution. This finding is contrary to that of Frascella et al. [49] who found that the use of email reminders improved immunisation uptake in their review. A contrary result was also shared by Balzarini et al. [50] who reported a moderate effect to exist for the use of personalized electronic health records for improved immunisation outcomes. Balzarini et al. [50] further reported that the effect is even more when the intervention was combined with digitalized health educational messages. From this, it could be deduced that when information communication gadgets are introduced into immunisation program implementations, it could improve outcomes by increasing vaccine use.

On the other hand, the use of SMS reminders to parents also showed promising results in improving coverage of immunisation with the odds of 1.7 times for the intervention compared to the control and was found to be statistically significant in producing a small effect based on Cohen’s scale [45]. Close observations to explain the disparity between the two significant findings reveals that of the nine studies used for the meta-analysis, only four [9,29,32,34] of the studies reported translating the messages sent to parents; the other five either used only English [26,44] or did not report translating [31,43]. This could have a significant effect on the outcome of individual studies. This position is supported by Cheung et al. [51], who reported the critical role of translating instruments for better validity.

Timely vaccination is increasingly seen as an essential indicator for preventing the needless death of children from vaccine-preventable death [9]. The meta-analysis of three methods was performed to determine the most effective method to enhance timely immunisation uptake. The interventions were: SMS reminders, incentives and the effect of health education delivered through SMS. The analysis indicates that immunisation educational messages sent to participants had more effect on the timely immunisation of children. This intervention produced the most significant effect for timely immunisation completion, indicating that participants engaged in this intervention are approximately 2.7 times more likely to complete their immunisation on time. Although this is significant, the effect size, when determined using Cohen’s scale [45], reveals a small effect. Although it was of small effect, it was however of high certainty indicating that the estimated results lie close to the true effect. Additionally, although it was found to have no significant heterogeneity and of high certainty, its use should also be used with caution because of the limited number of studies included in the meta-analysis. The finding corresponds to Galadima et al. [52], who found in their systematic review that health education to mothers on immunisation shows a significant effect on the immunisation outcome of children. To this end, evidence reveals that understanding risk associated with health care hesitancy of recommended counsel or intervention positively impacts service utilisation or compliance with clinical advice [53]. The result also supports the assertion of Balzarini et al. [50] who reveal in their systematic review that digitalised health education messages improve immunisation outcomes. It is also worth mentioning that the use of incentives also shows promising effects in this analysis. It could produce the desired effect if adequately harnessed, especially with increased incentive.

Other interventions not included in the meta-analysis were, using pendants with and without voice calls, home-based records with and without stickers, and Star and Alma Sana Bracelets. It is worth noting that some of these interventions significantly improved immunisation uptake but were not larger than the immunisation health information disseminated through mobile technology. Mobile technology has now opened up the space and offers an opportunity for improved communication between health practitioners and clients and is now being explored further for improved immunisation indicators [54,55]. Despite its demonstrated effectiveness here, it can be further enhanced to produce a larger impact by considering factors such as the appropriate timing, use of local languages to send messages and targeting the male partners in addition to women. Getting the male partner involved for improved immunisation outcomes is an assertion identified here that is yet to be explored.

Regarding cost implication, four studies reported the cost implication for the parental reminder. It is, however, essential to note that three of the four studies were conducted on cost analysis using the SMS reminder strategy in developing countries. A cost analysis of the studies indicates that, on average, USD 0.50 was used to implement an SMS call/recall intervention per child in LMICs. This finding proves that cheap and low-cost interventions can be implemented to improve immunisation coverage [56]. It is also worth noting that this intervention was found in this review to be effective with a moderate effect in improving immunisation coverage more than interventions with incentives. In addition, evidence reports that for every USD 1 spent to vaccinate a child, about USD 79 is saved [10]. This proves that SMS reminder/recall is a cost-effective intervention for improved immunisation coverage. However, using other technology (a centralised collaborative recall system) was found to cost an average of USD 10 for its intervention.

## 5. Implication

Policymakers can use the results of this review and apply them to their context by designing appropriate interventions such as health information using local languages for hard-to-reach populations to create more awareness and thereby reduce vaccine hesitancy. This can also help bridge the wide health inequality gap among the rural-urban population, thereby creating inclusion and increasing the use of vaccines.

## 6. Conclusions

This review was conducted to determine an effective strategy for enhanced timely immunisation and coverage. The findings of this review affirm the role of mHealth technology in delivering a cheap and efficacious health intervention for enhanced coverage and timeliness of immunisation. The study indicates that reminder voice calls and SMS-delivered health education improve immunisation outcomes. Though the effects of such interventions were moderate and small for coverage and timeliness, respectively, the researchers recommend that studies utilising mHealth should be explored more, especially on the influence of the male partners as a critical determinant to health service utilisation, especially in LMIC. Future studies should also consider determining the optimal dose required for the implementation of mHealth interventions for optimal vaccine coverage. Modules for mHealth health education could be developed that are population specific that can be used for mHealth educational interventions for improved vaccine use.

This study is limited because of the potential of excluding other studies for this review, such as articles not written in English, pilot studies and limiting the time for search to only eight years. Additionally, of the 24 included studies, only 5 were from HIC, and the other 19 were from LMICs; as such, interpretation of the findings in HICs should be made with caution. Due to heterogeneity and low certainty of evidence for intervention on improving coverage, the findings of the meta-analysis should also be interpreted with caution.

## Figures and Tables

**Table 1:** PICOS framework.

No.	Variable	Inclusion Criteria	Exclusion Criteria
1	Population	Parent of children	Assess immunisation other than in children less than five years
2	Intervention	Parental reminder strategies for immunisation	Intervention not targeting parents or caregivers of children less than five years
3	Comparator	Usual or standard care practice	
4	Outcome	Immunisation coverage, timeliness and cost of interventions	Outcomes other than coverage, timeliness and cost of interventions
5	Study design	Randomised or quasi-experimental studies	Survey, pilot study, non-peer review articles such as thesis

**Table 4:** Certainty of evidence for interventions on coverage of childhood immunisation.

Certainty Assessment							№ of Patients		Effect		Certainty	Importance
No. of Studies	Study Design	Risk of Bias	Inconsistency	Indirectness	Imprecision	Other Considerations	Incentive	Standard Care	Relative (95% CI)	Absolute (95% CI)		
Incentive on coverage of childhood immunisation												
3	Randomised trials	Serious ^a^	Not serious	Not serious	Not serious	None	1379/2884 (47.8%)	1508/2884 (52.3%)	OR 1.518 (1.238 to 1.862)	102 more per 1000 (from 53 more to 148 more)	⨁⨁⨁◯ Moderate	
SMS Reminder on coverage of childhood immunisation												
10	Randomised trials	Serious ^a^	Very serious ^c^	Not serious	Not serious ^d^	None	2683/5334 (50.3%)	2651/5334 (49.7%)	OR 1.671 (1.169 to 2.390)	126 more per 1000 (from 39 more to 206 more)	⨁◯◯◯ Very low	
Voice call on coverage of childhood immunisation												
4	Randomised trials	Serious ^a^	Serious ^c^	Not serious	Not serious	Publication bias strongly suspected ^e^ strong association	9597/19339 (49.6%)	9742/19339 (50.4%)	OR 4.752 (1.846 to 12.231)	325 more per 1000 (from 148 more to 422 more)	⨁⨁◯◯ Low	
SMS health education on coverage of childhood immunisation												
2	Randomised trials	Not serious	Serious ^f^	Not serious	Serious ^d^	None	1612/3247 (49.6%)	1635/3247 (50.4%)	OR 3.158 (0.301 to 33.121)	259 more per 1000 (from 270 fewer to 468 more)	⨁⨁◯◯ Low	
Voice call and SMS reminder on coverage of childhood immunisation												
2	Randomised trials	Serious ^a^	Serious ^g^	Not serious	Not serious	Strong association	601/1043 (57.6%)	442/1043 (42.4%)	OR 2.025 (1.211 to 3.389)	174 more per 1000 (from 47 more to 290 more)	⨁⨁⨁◯ Moderate	

CI: confidence interval; OR: odds ratio; ^a^ No blinding; ^c^ heterogeneity and inconsistency in effect sizes; ^d^ wide confidence interval in studies with few sample size; ^e^ as shown on funnel plot; ^f^ wide variance and inconsistency in effect size; ^g^ heterogeneity.

**Table 5:** Certainty of evidence for interventions on timeliness of childhood immunisation.

Certainty Assessment							No. of Patients		Effect		Certainty	Importance
№ of Studies	Study Design	Risk of Bias	Inconsistency	Indirectness	Imprecision	Other Considerations	Parental Strategies	Standard Care	Relative (95% CI)	Absolute (95% CI)		
Incentive on timeliness of childhood immunisation												
3	Randomised trials	Serious ^a^	Serious ^b^	Not serious	Not serious	Strong association	1031/1933 (53.3%)	902/1933 (46.7%)	OR 2.151 (1.613 to 2.867)	186 more per 1000 (from 119 more to 248 more)	⨁⨁⨁◯ Moderate	
SMS reminder on timeliness of childhood immunisation												
9	Randomised trials	Serious ^b^	Serious ^b^	Not serious	Not serious	Publication bias strongly suspected ^c^	2636/5248 (50.2%)	2612/5248 (49.8%)	OR 1.472 (1.164 to 1.863)	96 more per 1000 (from 38 more to 151 more)	⨁◯◯◯ Very low	
Health education on timeliness of childhood immunisation												
2	Randomised trials	Serious ^a^	Not serious	Not serious	Not serious	Strong association	198/401 (49.4%)	203/401 (50.6%)	OR 2.711 (1.387 to 5.299)	229 more per 1000 (from 81 more to 338 more)	⨁⨁⨁⨁ High	

CI: confidence interval; OR: odds ratio; ^a^ no blinding; ^b^ heterogeneity; ^c^ as shown on funnel plot.

## Data Availability

All data utilized for the purpose of this study are available publicly and online.

## Appendix A

**Table A1:** Table of data extraction.

No.	Author (Year)	Participants	Sample Size	Design	Intervention	Control	Vaccine type	Country	Dose		Instrument	Results
									Freq.	Course		
1	Niederhauser et al. [26]	Mothers	42	2-arm RCT	SMS reminder n = 19	Usual/standard care n = 23	DTaP, hepatitis B, Hib, PCV and polio	USA	2 SMS sent at 4 and 2 weeks to the due date	Seven months	Vaccination records	Immunisation coverage at seven months was 7 (41.2%) in the intervention group compared to 15 (65.2%) in the control group. This indicates that 58.8% and 34.8% were not up to date with immunisation at seven months.
2	Kempe et al. [28]	Mother–infant pair	18,235	RCT	Collaborative centralized reminder/recall approach (n = 9049)	Practice-based reminder/recall approach n = 9186	DPT, Poliovirus, measles, Hib, Hepatitis B, Varicella and PCV.	United States	Autodialled/mail protocol (2 calls or four mails followed by two postcards) Mail only (1 letter and three postcards every six weeks)	6 Months	Immunisation record	Intervention improves coverage by 26.9% for the collaborative centralized/recall approach treatment group compared to 21.7% for practice-based (*p* < 0.001).
3	Hofstetter et al. [39]	Mother–child pair	2054	RCT	A. scheduling + appointment SMS reminder (n = 686) B. appointment SMS reminder only n = 686	Usual care n = 682	MMR	United States	Three schedules plus one SMS	16 months	1. Hospital vaccination record 2. Vaccine cards	The intervention led to timely uptake of MMR vaccination 686(61.1%) vs. 682 (55.1%) (RRR 1.11 95% CI 1.01–1.21). For the SMS and control arm, respectively.
4	Bangure et al. [29]	Mothers	304	2-arm RCT	SMS reminder n = 152	Usual health talk n = 152	OPV, PCV and Pentavalent	Zimbabwe	Three times per visit on days 7, 3 and 1 before each scheduled	14 weeks	Immunisation register	On the 14th week, the intervention resulted in 82% of respondents receiving timely immunisation compared to only 8% in the control group. While at the 14^th^ week, vaccination coverage was 95% and 75% for the intervention and control groups, respectively.
5	Busso et al. [27]	Mothers	13,000	RCT	Community outreach with patients list requiring immunisation	Community outreach without patients list	BCG, Pentavalent, Poliovirus and MMR	Guatemala	Once a month	6 Months	Hospital records	Immunisation coverage increased by 4.6% among the intervention group The direct cost per child in the study was USD 0.11 per child for 6 months.
6	Brown et al. [30]	Mothers	595	RCT	A. Telephone call: reminder/recall n = 148 B. PHCIPT n = 150 C. Combination of A and B n = 147	C. Usual care practice n = 150	OPV, DPT, Hepatitis B, Measles and Yellow fever	Nigeria	A. Two reminder calls (on days 2 and 1 before due date) and recall four times B. Two days of one-off training for RI providers	12 months	American Immunisation Registry Association (AIRA) questionnaire	Coverage at the of the study was 98.6% for the voice call reminder or recall group compared to the control with coverage of 57.3%.
7	Uddin et al. [43]	Mothers	1030	Cluster quasi-experimental	Mobile SMS in rural areas n = 520	Mobile SMS in urban areas n = 518	BCG, Pentavalent and MR	Bangladesh	Three messages One a day to the scheduled visit, in the morning of the opening hour of the clinic and two hours to the close of the clinic	12 months	Immunisation register and mother’s recall	RURAL COVERAGE The intervention increased coverage in rural areas from 58.9% to 76.8%, difference +18.8% (95% CI 5.7–31.9) URBAN COVERAGE Urban coverage improved from 40.7% to 57.1% with a difference of +16.5% (95% CI 3.9–29.0).
8	Gibson et al. [31]	Mother	1600	4-arm cluster RCT	A. SMS-only n = 388 B. SMS+ 75 KES for each pentavalent received n = 446 C. SMS+ 200 KES for each pentavalent received n = 406	Usual care practice n = 360	BCG, OPV, Pentavalent and Measles	Kenya	SMS reminder on days 3 and 1 before the immunisation due date	12 months	Immunisation register	Full immunisation coverage at 12 months of age for SMS-only group, 296 (82%) of 360 control group compared to intervention participants, 332 (86%) of 388. RR = 1.04 (95% CI 0.97–1.12). SMS plus 75 KES intervention group had achieved 86% compared to the control group that had 82%, control participants group. Intervention comparing higher incentive 200 KES in addition to SMS leads to 90% 364 of 406 participants achieving immunisation coverage compared to the control group that had 82%, participants group. SMS plus 75 KES on the timeliness of immunisation is 70%; RR 1.37 (95% CI 1.18–1.59) compared to the control group of 50% timely coverage. SMS plus 200 KES on the timeliness of immunisation is 72%; RR 1.42 (95% CI 1.23–1.65) compared to the control group of 50% timely coverage.
9	Nagar et al. [7]	Mothers	198	3-arm RCT	A. Pendant with Voice Call arm (P + V). n = 75 B. Pendant only n = 61	Near Field Communication (NFC) stickers placed on the child immunisation card. n = 62	DTP	India	Voice call a day to due date and after the due date for no show	180 days from birth	Immunisation records and patient recall	DPT3 completion within 2 months. Control NFC sticker 74.2%, pendant only 67.2% and pendant +voice call 69.3. DPT Shots within 180 days 69.4, 57.4 and 58.7% for pendant with voice call, pendant only and NFC stickers, respectively.
10	Seth et al. [32]	Mothers	549	3-arm RCT	A. Automated SMS-only (n = 188) B. Automated SMS with airtime for each received scheduled vaccine n = 179	Immunisation record cards (n = 182)	OPV, Rotavirus, Pentavalent, PCV, IPV and MR	India	One (1) automated SMS sent before the scheduled due date	12 months	Immunisation records	Timeliness of immunisation dropped in the SMS-only participants by 24.7% (60 out of 243) when compared to the standard treatment group, 31.3% (76 of 243) Vaccination coverage for the control and intervention groups was 40.1% (Inter Quartile Range: 30.8–69.2%), and 50.0% (Inter Quartile Range: 30.8–76.9%), respectively. The incentive-linked group achieved immunisation coverage of 50.0% interquartile range (IQR: 30.8–76.9%) compared to the control group of 41.7% (IQR: 23.1–69.2%). 40.8% of the SMS and incentive intervention group completed immunisation on time compared to 31.3% of the control group.
11	O’Grady et al. [33]	Mother	196	3-arm RCT	A. Simple SMS. n = 64 B. educational SMS ± additional support group ESMS±S n = 65	Usual care practices n= 67	HepB-DTPa_Hib_IPV, PCV and Rotavirus	Australia	SMS sent 2 and 1 weeks before the due date and after two weeks if the child is still not vaccinated	Eight months	Questionnaire and immunisation record	Immunisation coverage rose to 70.3% of 45/64 in the SMS intervention group compared to 67.2% 45/67 in the control arm. At the end of the intervention, infants in the intervention group utilizing SMS-delivered educational messages had more vaccine coverage (83.1%) compared to the control group that received standard care 45/67 (67.2%).
12	Kazi et al. [39]	Caregiver	300	2-arm RCT	SMS reminder n = 150	Standard care practice n = 150	DPT-Hep-B-Hib vaccine OPV	Pakistan	Four SMS reminders sent on the week of the scheduled appointment	18 weeks	Record	14 weeks scheduled visit was 47 (31.3%) for the intervention group vs. 39 (26.0%) for the control group, *p* = 0.31). It is therefore not statistically significant.
13	Ekhaguere et al. [40]	Mothers	600	2-arm RCT	Voice call message and SMS n = 171	Standard care n = 140	Pentavalent, OPV, Rota vaccine, PCV, Measles and Yellow fever.	Nigeria	Two SMS or voice call at day two and one to the scheduled immunisation	12 month	Record	All scheduled immunisations collectively led to (57% vs. 47%, RR 1.13, 95% CI 1.02 to 1.26; *p* = 0.01) within 1 week of the recommended date for the intervention compared to the control group. The cost of delivering the SMS was USD 0.0075 and USD 0.015 for SMS and voice call, respectively.
14	Dissieka et al. [41]	Mothers	1596	2-arm RCT	Voice call and SMS n= 484	Standard care n = 302	Pentavalent, MMR and Yellow fever immunisations	Côte d’Ivoire	One SMS reminder and call and two recalls	9 month	Records	Immunisation coverage at 9 month were 484 (60.7%) for the intervention group compared to 302 (37.8%) for the control with the adjusted odds ratio of 4.52 (2.84–7.20).
15	Domek et al. [34]	Mothers	720	RCT	SMS sent in local languages n = 329	The usual practice of care using child card n = 333	Pentavalent, PCV, IPV, OPV, Rotavirus	Guatemala	3 SMS at 3, 2 and 1 day to the due date	Eight months	Electronic immunisation record	Timeliness at Visit 3 was found to be 112 (34.0%) of 329 for experimental group and 90 (27.0%) of 333 in the control group, *p* = 0.05. Both intervention and control groups had a high rate of immunisation coverage (89.1% vs. 89.2%) for intention to treat analysis but were not statistically different.
16	Wallace et al. [12]	Mothers	3616	Cluster RCT	A. Home base records (HBR) only. N = 1290 B. Home base record + stickers. n = 711	Routine care practice n = 1615	DPT	Indonesia	A. HBR provided whenever, missing, damaged or destroyed and information transferred to the new one. B. In addition to A above, stickers are placed indicating due date for each immunisation visit.	7 months		HBR + sticker group for DTPcv3 vaccination completion rate (77%) compared with the control group (81%) (RR = 0.97, 95% CI:0.90, 1.04), neither HBR-only group for DTPcv3 (74%) compared with the control group (RR = 0.94,95% CI:0.87, 1.02). HBR + sticker vs. control (77 vs. 78%) (RR = 0.99, 95% CI: 0.98, 1.09), HBR-only vs. control 74% (RR = 0.96, 95% CI: 0.88, 1.05) However, children in the HBR + sticker group were 50% more likely to.
17	Menzies et al. [35]	Mothers	1594	Four arm RCT	A. SMS-only. (n = 398) B. Personalized calendar (PC) only. n = 398 C. PC+ SMS. n = 404	Usual care practice n= 394	DPT, Polio, Hib, Rotavirus, Pnuemococcal, MMR and Varicella	Australia	2 SMS at 14 and 2 days to the due date	Ten months	Excel spreadsheet information extracted from immunisation register	Results on the 6th-month timely immunisation show 310 (78%) RR 1.05 (95% CI 0.97–1.14) SMS reminder intervention group had their children immunised on time compared to 291 (74%) of the control arm.
18	Brownstone et al. [36]	Mothers	951	3-arm RCT	Model 1: higher incentive (baseline amount NGN 3000 + NGN 1000 a reminder call) n = 345	Model 2: more minor incentive (baseline amount NGN 2000 + NGN 1000 plus a reminder call). n = 606	BCG, Pentavalent, PCV and Measles	Nigeria	One call or SMS before the scheduled date and an additional increase in incentive	Four months	Child health record book	Results from the intervention show that increasing monetary intervention was effective in resulting in 90.1% coverage of measles vaccine compared to 86.1% recorded in the control group.
19	Siddiqi et al. [37]	Caregiver	1445	3-arm RCT	A. Alma Sana Bracelet Group n = 482 B. Star Bracelet Group n = 482	Usual care practice n = 481	Pentavalent and Measles	Pakistan	6 punctures at the appropriate age or 6 crescent punctures before completion of vaccination. One per visit	12 months	Hospital records	Coverage of Penta 3 at 12 month is 84.3, 85.4, 83% for Alma Sana Bracelet, Star Bracelet and control group, respectively. While measles 1 coverage at 12 months was 72.0, 70.5 and 68.5% for Alma Sana Bracelet, Star Bracelet and control group, respectively.
20	Mekonnen et al. [9]	Mothers	426	2-arm RCT	Reminder SMS n = 213	Usual care practice n = 213	BCG, OPV, Rotavirus, Pentavalent, PCV, Rotavirus, Measles and Inactivated Polio	Ethiopia	1 SMS sent a day to the due date	12 months	Hospital record	Timely vaccination was 213 (63.3%) and 213 (39.9%) for experimental and control group, respectively *p* < 0.001; risk ratio 1.59, 95% lower CI: 1.35. While coverage was 82% of 176/213 compared to 70.9% of 151/213, respectively; *p* = 0.002; Risk Ratio 1.17, 95% lower CI 1.07) compared to those in the control group.
21	Kagucia et al. [38]	Caregivers	537	3-arm RCT	A. SMS reminder n = 146 B. SMS reminder+ monitory incentive (150KES) n = 149	Usual care practice n = 160	Measles vaccine	Kenya	2 SMS sent 3 and 1 day to the due date	3 months	Checklist and questionnaire	SMS intervention yielded 146 (78%) timely completion against 160(68%) timely coverage in the control arm with adjusted RR 1.13; 95% CI 0.99 1.30; *p* = 0.070.
22	Ibraheem et al. [44]	Mothers	540	4-arm Quasi-experimental	A. SMS reminder n = 136 B. Phone call reminder n = 133 C. SMS health education n = 133	Routine care practice n = 136	Pentavalent, PCV, OPV, IPV, Measles and Yellow fever	Nigeria	1 SMS a day to the due date	Five months	Immunisation register	86 (63.7%) received timely the 9th-month immunisation schedule while the 45 (36.6) vaccines timely for the intervention and control arm, respectively, *p* < 0.001. Completion of immunisation at 9 months was higher among the SMS reminder group (99.3%) compared with the control group, with percentage coverage of 90.4%. Immunisation fact messages sent through SMS improved immunisation timeliness in the intervention group compared to the control group of 97% and 90.4%, respectively. SMS conveyed health education messages yield 99.2% immunisation coverage compared to 90.4% in the standard care group. Coverage for phone call was 99.2% compared to control with coverage of 90.4%.
23	Oladepo et al. [13]	Mother–infant pair	3139	2-arm quasi experimental	SMS on immunisation health education n = 1479	Flyers on nutrition and growth monitoring n = 1499	BCG, Pentavalent, OPV, HBV, IPV, Measles and Yellow fever	Nigeria	Immunisation education messages are sent three times a week	Ten month	Immunisation register	SMS education messages led to a 76.0 vs. 73.3% completion rate in the control group with standard care.
24	Yunusa et al. [15]	Mothers	541	2-arm Quasi experimental	Mobile phone call reminder n = 271	Routine care practice with child health card n = 270	Pentavalent	Nigeria	Two calls on days 3 and 1 and call if the client fails to show up	Four month	Immunisation register	The coverage rate in the experimental group was 59.4% compared to the control group of 34.1% after four months of intervention.

Key: HepB-DTPa_Hib_IPV = (hepatitis B, diphtheria, tetanus, acellular pertussis (whooping cough), Haemophilus influenzae type B, polio), MMR = Measles–mumps–rubella, IPV = Inactivated polio vaccine, OPV: Oral polio vaccine, DPT = Diptheria pertussis tetanus, BCG = Bacillus Calmette–Guérin vaccine against tuberculosis, MR = Measles–Rubella, PCV = Pneumococcal conjugate vaccine, SMS = Short Message Services, HBR = Home-Based Record, RCT = Randomised control trials, ESMS = Educational Short Message Services.

**Table A2:** Cohen’s Table of Effect Size.

	Trivial	Small	Moderate	Large	Very Large	Nearly Perfect	Perfect
Correlation	0.0	0.1	0.3	0.5	0.7	0.9	1
Difference in means	0.0	0.2	0.6	1.2	2.0	4.0	Infinite
Frequency difference	0	10	30	50	70	90	100
Relative risk	1.0	1.2	1.9	3.0	5.7	19	Infinite
Odd ratio	1.0	1.5	3.5	9.0	32	360	Infinite
